# A New Feature

**Published:** 1859-01

**Authors:** 


### A NEW FEATURE.

We shall commence in our next issue, the report of the College Clinic, which has been in operation since the commencement of the Winter exercises in the Atlanta Medical College. Since the conclusion of the last regular course of Lectures, arrangements have been made with the City Authorities, by which a dispensary is established at the College. In this, patients are daily examined and prescribed for by the Professors of the Institution, and instead of a tri-weekly Clinic as heretofore, the Students have the advantages of witnessing these investigations every day.

We hope these reports, which will be prepared monthly for the Journal, will be found interesting to our readers.

The reports will include the progress of the cases presented, whether the plan of treatment prove successful or otherwise, and the changes of remedies used, and the result of such change.

To the Students of Medicine, who may favor us with their presence during our Regular Course next Summer, the Clinic promises much, and to those now in attendance upon the Preparatory Course, this plan of Clinical instruction is proving highly useful We are glad to know that the greatest interest and satisfaction is manifested by the young gentlemen in attendance upon our daily Clinics this Winter.

With the advantages afforded by the warmer season, for increasing the number of cases, we hope to add still to the interest of the regular Course, and to the usefulness of the reports to the readers of the Journal.

Greatly as we value the advantages of large Hospitals, well stored with invalids, to the Student of Medicine, who can devote the necessary time and attention to their consideration, we are thoroughly convinced that one hour per day occupied in these practical demonstrations before the whole Class, gives them more useful information, than half a dozen hospitals, by the imperfect exhibition of the patients in their wards to the crowded class, at the *heels* of the physician in attendance. Some of the peculiarities of an unfortunate case may be caught by the more fortunate, but no perfect understanding of the description and treatment of the diseases, or of their progress from time to time. To do this, the Student must see them regularly, and patiently note the symptoms, each time comparing those present, with the condition of the previous examination. At the same time making himself familiar with the modes of physical and other examinations, practically. In this way much useful and practical information may be obtained from extensive hospitals. But while in attendance upon a regular Course of Lectures in a Medical College, few, if any, find time and opportunity to pursue this course. Most of the time, aside from that occupied in reading, must be spent in the hospital. Therefore the advantages of hospital investigations cannot be had in connection with the benefits of College Lectures. One of these must be taken separately, in order to realize its advantages.

If it is desired by the Student, to make himself familiar with the nature and treatment of disease practically, in this way, before he has completed his Collegiate Course, he should do so by postponing for the time, his attendance upon a Course of Lectures.

It will thus be perceived that our motto is, "Excelsior," and that Faculty of the Atlanta Medical College, are constantly adding to their means of instruction, and are industriously preparing for the interest and profit of the large Class, which we are assured will assemble here at the next Session, commencing on the 1st of May, 1859.

